# Research status and hotspots in the surgical treatment of tremor in Parkinson’s disease from 2002 to 2022: a bibliometric and visualization analysis

**DOI:** 10.3389/fnagi.2023.1157443

**Published:** 2023-09-27

**Authors:** Jingchun Zeng, Hui Chu, Yiqian Lu, Xi Xiao, Liming Lu, Jingjing Li, Guoan Lai, Lisha Li, Lihong Lu, Nenggui Xu, Shuxin Wang

**Affiliations:** ^1^Rehabilitation Center, The First Affiliated Hospital of Guangzhou University of Chinese Medicine, Guangzhou, China; ^2^The First Clinical Medical College of Guangzhou University of Chinese Medicine, Guangzhou, China; ^3^Clinical Research and Data Center, South China Research Center for Acupuncture and Moxibustion, Medical College of Acu-Moxi and Rehabilitation, Guangzhou University of Chinese Medicine, Guangzhou, China; ^4^Bao’an Traditional Chinese Medicine Hospital, Seventh Clinical Medical College of Guangzhou University of Traditional Chinese Medicine, Shenzhen, China; ^5^Xingtan Hospital, The Affiliated Shunde Hospital of Southern Medical University, Foshan, China

**Keywords:** tremor, Parkinson’s disease, surgical treatment, visualization, research hotspots

## Abstract

**Objective:**

This study aims to investigate the research status and hotspots of surgical treatment for tremor in Parkinson’s disease (PD) from 2002 to 2022, utilizing bibliometric and visual analysis. Additionally, it aims to offer insights into future research trends in this field.

**Methods:**

This study collected publications on the surgical treatment of tremor in PD from 2002 to 2022 using the Web of Science (WOS) database. CiteSpace, VOSviewer, and Scimago Graphica were employed to quantify the number of publications and analyze the bibliographic information networks, including the contributions of countries/cities, authors, keywords, and co-cited references.

**Results:**

A total of 2,815 publications were included in the study, revealing that 541 scientific institutions experienced an increase in publications from 2002 to 2022. Michael Okun emerged as the most productive author, and the United States emerged as the leading hub for research. The study identified 772 keywords. Noteworthy citation bursts and long-term activity were observed in pallidotomy, bilateral stimulation, and focused ultrasound thalamotomy. The top 10 highly co-cited references comprised eight deep brain stimulation (DBS) studies (including two follow-up studies and six randomized controlled trials), one randomized controlled trial on focused ultrasound, and one consensus on tremor.

**Conclusion:**

This study uses an in-depth and systematic bibliometric and visualization analysis to visualize the evolution of research and identify emerging hotspots. The identified hotspots are as follows: Firstly, DBS has received significant attention and widespread recognition as a surgical treatment for tremor in PD. Secondly, there are various key aspects to consider in DBS, such as operative indications, operative targets, and surgical protocols. Lastly, magnetic resonance-guided focused ultrasound (MRgFUS) has emerged as a promising treatment option in the surgical management of tremor in Parkinson’s disease. This research also provides insights into the phenomenon of these hotspots, offering valuable prompts and reminders for further research.

## 1. Introduction

Parkinson’s disease (PD) is a chronic and progressive complex neurodegenerative disorder characterized by the core motor symptom of tremor. Although drug therapy is the primary treatment for PD (Hayes, 2019), its efficacy diminishes due to the disease’s gradual progression and unpredictable adverse effects, making surgical intervention a consideration. Current surgical modalities for PD tremor can be primarily classified into neuromodulation and neurodestructive procedures. Neuromodulation comprises spinal cord stimulation and deep brain stimulation (DBS) (Merola et al., 2021). DBS, approved by the Food and Drug Administration (FDA) in 2002 for PD treatment, involves the stimulation of specific deep brain nuclei using different chronic microcurrents. The procedure comprises two parts: implantation of the DBS device and post-operative program control. Its mechanism of action is complex, involving hypotheses like the interruption, inhibition, and excitation hypotheses, which are interrelated and vary in importance based on treatment conditions and stimulation target.

However, neurodestructive procedures mainly encompass magnetic resonance-guided focused ultrasound (MRgFUS), magnetic resonance-guided laser interstitial thermotherapy, and radiofrequency ablation (Harris et al., 2019; Garcia-Gomar et al., 2020; Yamamoto et al., 2021). Due to the post-operative complications associated with traditional radiofrequency ablation and advancements in imaging and ultrasound technology, novel neuron-destruction procedures, like magnetic resonance-guided laser interstitial thermotherapy and MRgFUS, have emerged. MRgFUS received FDA approval in 2019 for PD treatment. Surgical modalities for PD also encompass gene therapy and cell transplantation, alongside spinal cord electrical stimulation, currently under clinical research. However, there have been no studies reporting significant efficacy for PD tremor as of yet. Therefore, it is urgent to investigate the research status and hotspots of surgical treatment for tremor in Parkinson’s disease (PD) from 2002 to 2022.

## 2. Materials and methods

### 2.1. Search strategy and data collection

We conducted a search in the WOS database on August 31, 2022, to identify relevant studies evaluating surgical interventions for tremor in PD published from 2002 to 2022. The search terms were based on medical subject headings (MESH) and synonyms of relevant text terms, including (Parkinson*) and (*tremor* or pill-rolling), and (*surgery* or surgical* or *stimulation or Radiofrequency ablation or MRI-guided laser interstitial thermal or MRI-guided focused ultrasound or stereotactic radiotherapy or Gamma knife thalamotomy or cell transplantation therapy or gene therapy). Literature-type language was not restricted, and the search period was set from January 1, 2002, to December 31, 2022. We used CiteSpace to remove duplicate publications, resulting in 2,815 unique articles. The search process is illustrated in Supplementary Figure 1.

### 2.2. Bibliometric and visual analysis

A Java-based visualization program called CiteSpace software, designed by Professor Chen Chaomei from Drexel University in the United States, utilizes the theory of co-citation (Chen, 2004; Chen and Song, 2019). In this study, we employed CiteSpace software (v6.1.R2, v6.1.R3), VOSviewer (v1.6.18), and Scimago Graphica (v1.0.25) to conduct a scientometric analysis. The dataset was managed using Microsoft Excel (2018), with duplicates and rejects removed before applying CiteSpace for data plotting, keyword co-occurrence analysis, and co-citation analysis. Additionally, VOSviewer (v1.6.18) was utilized to map cooperation networks (authors, institutions, and countries), while Scimago Graphica (v1.0.25) was employed for country/region analysis and to visualize the number of publications in different regions and countries.

CiteSpace parameters were set as follows: time slice: 1 year; period: January 2002 to December 2022; term source: all items, one node type at a time; other settings were set to default values.

### 2.3. Interpretation of the visualization map

The knowledge maps contain nodes and links. The round areas, termed “citation nodes,” represent the citation history of a paper. The node size indicates the frequency of varied items (author, Country/region, Institution and so on) and is positively correlated with the number of references. The links between nodes signify the strength of cooperation, co-occurring keywords, and co-citation relationships. Bursts mean that the value of a variable has changed significantly in a short period of time, which also indicates that the variable is an important turning point in the field of study, which is worth paying attention to. The Burst analysis not only identifies papers attracting more attention from peer researchers, but also helps discover articles with significant influence on future research promptly. The centrality is a key indicator to the importance of keywords. If the centrality is more than 0.1, it means that the node is a central node, which is more important and influential in the study. The strength value reflects the citation frequency. The blue blocks represent the years 2002 to 2022, while the red blocks indicate the beginning, end, and duration of citation bursts. Clustering#: Each cluster consists of closely related keywords (each tag can be divided into several categories, e.g., #0, #1, #2……). The smaller the serial number, the more keywords its cluster contains—timeline: Each cluster is displayed as a horizontal timeline in a left-to-right order. The size of the circles on the map represents the number of citations. The timeline graph also reflects the time interval between 2002 and 2022 when the citation was noticed (Chen, 2017; Chen and Song, 2019).

## 3. Results

### 3.1. Annual publication volume

A total of 2,815 publications were identified in the WOS database for the period from 2002 to 2022 (Supplementary Figure 2).

### 3.2. Authors

The study identified 322 authors. Among them, Michael Okun (*n* = 83), Andres Lozano (*n* = 50), and Kelly Foote (*n* = 31) were the top three authors in terms of the number of articles (Supplementary Figure 3).

### 3.3. Country/region

The study involved researchers from 43 countries/regions. Table 1 presents the contributions from each country, with the United States leading with the highest number of publications (*n* = 1,204; 42.8%), followed by Germany (*n* = 400; 14.2%), the United Kingdom (*n* = 279; 10.0%), Canada (*n* = 221; 7.9%), and China (*n* = 204; 7.2%) (Table 1 and Supplementary Figure 4).

**TABLE 1:** Top 10 countries/regions by publications.

Rank	Countries/ Regions	Publications	Percentage (total of 2,815; %)
1	United States	1204	42.8
2	Germany	400	14.2
3	United Kingdom	279	10.0
4	Canada	221	7.9
5	China	204	7.2
6	Italy	178	6.3
7	France	139	4.9
8	Japan	129	4.6
9	Netherlands	116	4.1
10	Spain	109	3.9

### 3.4. Institution

Out of 541 scientific institutions, seven published more than 50 papers each, namely, the University of Toronto, University of Florida, University of Oxford, Mayo Clinic, Emory University, University of California San Francisco, and Baylor College of Medicine (Table 2 and Supplementary Figure 5).

**TABLE 2:** Top 10 institutions according to publications.

Rank	Publications	Institutions	Original country
1	128	University of Toronto	Canada
2	111	University of Florida	United States
3	78	University of Oxford	England
4	59	Mayo Clinic	United States
5	57	Emory University	United States
6	54	University of California San Francisco	United States
7	50	Baylor College of Medicine	United States
8	49	University of Kiel	Germany
9	48	University College London	United States
10	48	Duke University	United States

### 3.5. Co-occurring keywords

The study involved 772 keywords, with the top 10 most frequent ones being essential tremor (*n* = 671), movement disorder (*n* = 424), tremor (*n* = 375), basal ganglia (*n* = 370), globus pallidus (*n* = 289), subthalamic nucleus stimulation (*n* = 280), and thalamic stimulation (*n* = 254) (Supplementary Figure 6).

Clustering analysis revealed 10 co-occurring keyword clusters: #0 basal ganglia, #1 deep brain stimulation, #2 quality of life, #3 zona incerta, #4 high frequency stimulation, #5 orthostatic tremor, #6 blood-brain barrier, #7 double-blind, #8 machine learning, #9 tremor suppression, and #10 transcranial magnetic stimulation (Supplementary Figure 7).

A timeline map was constructed for the co-citation graph based on the time nodes. Supplementary Figure 8 displays the top three keywords with the highest level of activity and increasing attention since 2002: #0 basal ganglia, #1 deep brain stimulation, and #2 quality of life (Supplementary Figure 8).

The top 10 keywords with the most powerful citation bursts were: pallidotomy (12), thalamotomy (9.59), bilateral stimulation (13.43), multicenter (10.25), focused ultrasound thalamotomy (9.57), DB (9.53), Parkinson (10.04), consensus statement (8.78), s disease (11.16), and functional neurosurgery (9.57) (Supplementary Figure 9). Since our research theme was surgical treatment of PD we excluded Parkinson (10.04), consensus statement (8.78). We also excluded keywords with poor spelling, including DB (9.53), s disease (11.16).

### 3.6. Co-citations reference

Supplementary Figure 10 displays 1,467 nodes, and Table 3 lists the top 10 references with high co-citations. The table provides a summary of essential information, including the number of citations, journal, study type, sample size, patients, interventions, outcomes, and highlights for each reference (Schuurman et al., 2000; Deep-Brain Stimulation for Parkinson’s Disease Study Group et al., 2001; Krack et al., 2003; Deuschl et al., 2006; Follett et al., 2010; Little et al., 2013; Schuepbach et al., 2013; Elias et al., 2016; Cury et al., 2017; Bhatia et al., 2018).

**TABLE 3:** Top 10 highly cited references details.

References	Citation counts	Journal	Design or type of study	Sample size	Participants	Intervention	Outcomes	Highlights
Elias et al., 2016	109	*NEJM*	Single-blind, randomized, controlled trial	76	Patients diagnosed with essential tremor, with moderate to severe postural or intentional tremor of the hand, who have failed at least two trials of drug therapy and whose current drug dose is stable for primary tremor.	MRgFUS vs. sham procedure.	The Clinical Rating Scale for tremor and the Quality of Life in Essential tremor Questionnaire	1. Demonstrated the efficacy of FUS in drug-refractory primary tremor. 2. Long-lasting efficacy (12 months)
Bhatia et al., 2018	97	*Movement Disorders*	Consensus	\	\	\	\	1. Classification of tremor based on two axes. 2. To provide a framework for clinical diagnosis of new tremor syndromes for further etiological studies.
Krack et al., 2003	79	*NEJM*	Follow-up study	49	Patients aged less than 70 years with advanced PD after bilateral stimulation of the subthalamic nucleus	Bilateral STN-DBS	UPDRS, Mattis Dementia Rating Scale, an assessment of frontal-lobe dysfunction, Beck depression inventory	1. Demonstrated that STN-DBS significantly improved all post-operative PD motor symptoms. 2. No stimulus tolerance was found (5-year follow-up). 3. It is proposed that the procedure is more effective in patients with PD dyskinesia who respond well to medication and are relatively young.
Little et al., 2013	78	*Annals of Neurology*	Randomized controlled study	8	Patients with advanced idiopathic PD with motor fluctuations and/or dyskinesias	aDBS vs. no stimulation vs. cDBS.	UPDRS	1. Optimize the DBS stimulation protocol. 2. Design a BCI-controlled adaptive DBS (aDBS).
Schuepbach et al., 2013	77	*NEJM*	Randomized, multicenter, parallel-group	251	PD patients with early motor complications	DBS plus medical therapy or medical therapy alone	UPDRS, the time with good mobility and no dyskinesia.	Neurostimulation was indicated for earlier, younger patients (mean duration of disease of 7.5 years; mean age of 52 years; fluctuations and motor deficits for 1.7 and 1.5 years, respectively).
Deuschl et al., 2006	71	*NEJM*	Non-blind, randomized, paired trial	156	Patients younger than 75 years with impaired motor symptoms of PD affecting the ability to perform daily living with optimal drug therapy.	DBS group vs. drug group	PDQ-39, UPDRS	1. Used quality of life as the primary outcome indicator 2. The risks of surgery need to be weighed against the benefits of surgery.
Schuurman et al., 2000	70	*NEJM*	Randomized controlled trial	68	Severe unilateral or bilateral arm tremor due to PD, primary tremor, or multiple sclerosis for at least one year in the case of medication.	Thalamotomy or thalamic stimulation	Frenchay Activities Index, UPDRS, Essential tremor Rating Scale, Modified Tremor Scale.	1. DBS is more effective and safer than thalamotomy. 2. STN is a superior target than thalamus.
Deep-Brain Stimulation for Parkinson’s Disease Study Group et al., 2001	69	*NEJM*	Prospective, double-blind, crossover study	134	Patients aged 30–75 years with at least two major features of PD (tremor, rigidity, and bradykinesia) that are not controlled by medication and respond well to levodopa	Randomly turn on/off the DBS to STN or globus pallidus (GPi)	UPDRS, a dyskinesia-rating scale, a home diary	1. Demonstrated the effectiveness and safety of two DBS targets. 2. STN may be superior to the pallidum as a target.
Cury et al., 2017	65	*Neurology*	Follow-up study	98	PD, ET, and dystonia due to refractory tremor	VIM-DBS	UPDRS; Fahn, Tolosa, Marin tremor Rating Scale	1. Long follow-up period (over 10 years) 2. VIM is effective for PD tremor in the long term but does not delay PD progression. 3. VIM is the preferred target for tremor.
Follett et al., 2010	65	*NEJM*	Multi-center, randomized, blinded trial	299	PD patients over 21 years of age with Hoehn Yahr score greater than 2 who are stable on medication and have poor efficacy	Pallidal DBS or STN- DBS	Hoehn and Yahr scale, Schwab and England scale of activities of daily living, stand–walk–sit test; UPDRS, PDQ-39, Beck depression inventory-II.	1. Demonstrated the efficacy of DBS in the two target areas and the reduction of dopaminergic medication. 2. Improvements in pulse generators can reduce the impact of amplitude in DBS surgery.

## 4. Analysis of results

### 4.1. Analysis of annual publications, countries/regions, institutions, authors, and co-cited authors

This study focused on publications related to the surgical treatment of tremor in PD between 2002 and 2022. A bibliometric analysis was performed using the WoS database to gain insights into research dynamics and facilitate future investigations. A total of 2,815 articles were included in the analysis. Over the specified period, there was a consistent increase in annual publications, indicating a growing interest in and efforts toward the surgical treatment of tremor in PD. The United States emerged as the leading contributor, with the highest number of publications and citations. In the country/region co-authorship analysis network, the United States ranked first, underscoring its significant influence and prominent role in research on the surgical treatment of PD tremor. Remarkably, seven out of the top 10 institutions conducting research in this field were based in the United States. These results suggest that the United States may have substantial influence and play a leading role in the direction of research on the surgical treatment of PD tremor. Through a comprehensive examination of prolific authors and their articles, current research hotspots in the surgical treatment of PD tremor were identified. Notably, Michael Okun (*n* = 83) from the University of Florida was the most prolific author, followed by Andres M Lozano (*n* = 50) from the University of Toronto, and Kelly D Foote (*n* = 31) from the University of Florida. Highly productive authors could reflect the current research frontiers in the field. The research areas of the highly productive authors also verify the reliability of citespace’s hotspot results. Therefore, we have summarized the research directions of Michael Okun, Andres M Lozano and Kelly D Foote. Specifically, Michael S. Okun and Kelly D Foote, both affiliated with the University of Florida, co-founded and co-direct the renowned Norman Fixel Institute for Neurological Diseases at UF health. Their focus on DBS and neuromodulation has demonstrated that DBS is a practical surgical approach for treating tremor. Dr. Andres M Lozano focuses on functional neurosurgery and the development of new therapies for the treatment of movement disorders and psychiatric disorders. His team conducts research on brain mapping, DBS, and focused ultrasound (FUS) in both patients and animal models of the disease.

### 4.2. Analysis of co-occurring keywords

Keywords play a crucial role in reflecting the themes and frontiers of research articles. Through the co-occurring keywords clustering analysis, we observed a strong focus on tremor in the context of the surgical treatment of PD. Notably, the subthalamic nucleus and DBS emerged as prominent research hotspots. The clustering of keyword modules, as indicated by Modularity Q (>0.3) and Mean Silhouette (>0.7), is highly convincing. In the co-occurring keyword time zone map, #0 basal ganglia and #1 DBS were found to be actively studied, and they garnered sustained attention over an extended period.

Citation burst identifies keywords with high frequencies at specific times, indicating the evolutionary trends in a particular study. Notably, pallidotomy (burst = 12; 2002–2010), bilateral stimulation (burst = 13.43; 2004–2010), and focused ultrasound thalamotomy (burst = 9.57; 2017–2022) exhibited prolonged periods of continuous research focus. Furthermore, tracing back, we observed a renewed interest in pallidotomy in 1992 after a dormant period of PD tremor surgery, followed by a remarkable surge in pallidotomy-related publications (Laitinen et al., 1992; de Bie et al., 1999; Hariz, 2003; Vitek et al., 2003).

During this period, DBS surgery emerged, and related research rapidly developed. During the early 20th century, numerous studies confirmed the efficacy of bilateral DBS (Limousin et al., 1995; Esselink et al., 2004; Smeding et al., 2005; Gross, 2008). In 2011, the World Movement Disorders Society guidelines reviewed reports on motor symptoms in PD from 2004 to 2010, ranking bilateral subthalamic nucleus DBS, bilateral pallidum DBS, and unilateral pallidotomy as the highest level of evidence (Fox et al., 2011). Concurrently, advancements in technology enabled the use of phased-array transducers to deliver precise, incisionless, transcranial acoustic energy, leading to the development of focused ultrasound (FUS) for lesion destruction. Consequently, several studies explored the application of FUS in various brain diseases, and in 2017, a randomized controlled trial (RCT) by JAMA Neurol provided further evidence that ultrasound-focused thalamotomy effectively relieved tremor in drug-resistant PD, a significant milestone (Martin et al., 2009; Elias et al., 2016; Bond et al., 2017). Technological advancements continued, and in 2019, the FDA approved MRgFUS for the treatment of tremor in PD and primary tremor, marking a significant advancement in the field.

The co-occurring keyword analysis reveals essential themes in the research on the surgical treatment of tremor in PD. Notably, neuron destruction procedures, including pallidotomy, thalamotomy, and focused ultrasound thalamotomy, along with neuromodulation techniques like DBS, subthalamic nucleus stimulation, and thalamic stimulation, emerge as significant areas of interest. Moreover, studies of the brain regions involved in Parkinson’s disease tremor, such as the subthalamic nucleus, basal ganglia, and globus pallidus, also hold crucial importance in this field.

### 4.3. Analysis of top 10 highly co-cited references

Highly co-cited references are regarded as seminal contributions in the field and represent articles with significant influence. The frequency of citations indicates the literature’s impact. Among these references, seven were clinical trials, two were follow-up studies, and one was a consensus statement. The top 10 most frequently cited references provide insights into the key topics that have garnered researchers’ attention in the surgical treatment of PD tremor and reveal essential research areas in this field. Among these references, Elias et al. (2016) stands out as the most frequently co-cited, with a centrality value of 0.03. This paper focuses on MRI-guided focused ultrasound technology (MRgFUS), which has emerged as a prominent area of research in recent years, presenting new hotspots and trends that warrant attention. Notably, the remaining nine references are DBS-related, highlighting the current significant interest in DBS among researchers. By analyzing these eight papers, we identified three primary areas related to DBS research: (1) operative indications, encompassing timing, purpose, post-operative adverse effects, and prognosis of the surgery; (2) exploration of new targets; and (3) advancements in DBS devices.

## 5. Discussion

### 5.1. Bibliographic characteristic

This discussion centers on the research status and hotspots concerning the surgical treatment of tremor in PD from 2002 to 2022. We collected a total of 2,815 relevant publications from the WOS database and primarily analyzed them using CiteSpace software.

The analysis of relevant publications revealed insightful findings from diverse perspectives. Firstly, a notable upward trend was observed in the number of publications, indicating an increasing focus on the surgical treatment of tremor in PD. Secondly, the geographic distribution of publications showed early research initiatives from European countries (Germany, the United Kingdom), Canada, and China, while the United States emerged as the leading contributor, with eight out of the top 10 institutions that produced the most publications situated in the country. These outcomes strongly suggest the prominent role of the United States in this field. We should put more attention to the research from the United States.

The top three authors in terms of the number of articles and co-cited authors were Michael Okun, Andres Lozano, and Kelly Foote, showcasing their significant contributions to the field of surgical treatment of tremor in PD. Particularly, Andres Lozano’s article on a randomized trial of focused ultrasound thalamotomy, published in the New England Journal of Medicine, held the first position among co-cited references, underscoring the pivotal role of MRgFUS in the surgical treatment of tremor in PD. Researchers could start from the works of these prolific authors in order to know the hotspots in the field of surgical treatment of PD tremor, and could also grasp the frontier directions in the field of surgical treatment of PD tremor by studying the research directions of these prolific authors.

Keywords co-occurrence and co-occurring keywords clustering analysis have enabled us to rapidly and directly identify the general themes and hotspots in the field of surgical treatment of tremor in PD. In our analysis, the subthalamic nucleus and DBS garnered significant attention from researchers. Meanwhile, the basal ganglia and DBS remained active subjects of longstanding interest. This observation indicates that DBS and operative targets represent prominent hotspots in the field of surgical treatment of tremor in PD. Initially, early researchers primarily focused on pallidotomy, but as time progressed, there was a gradual shift toward a safer and more effective alternative—DBS, which has maintained sustained attention over the years. Moreover, recent advancements in non-invasive nerve destruction techniques have brought focused ultrasound thalamotomy to the forefront as a new and promising hotspot in the field of surgical treatment of tremor in PD.

In our investigation of co-cited references, we identified the top 10 highly co-cited articles, recognized as significant landmarks in the field of surgical treatment of tremor in PD. We then conducted a detailed examination of these 10 publications. Notably, eight out of the 10 references were dedicated to DBS, highlighting its pivotal role in the field of surgical treatment of tremor in PD. These references not only underscore the importance of DBS but also shed light on several crucial research areas and key aspects related to DBS. Accordingly, we meticulously analyzed these eight references to gain insights into the overall landscape of DBS in the context of surgical treatment for tremor in PD.

The eight references acknowledged the efficacy of DBS and primarily addressed three crucial aspects within the realm of surgical treatment for tremor in PD: operative indication (encompassing timing, purpose, post-operative adverse effects, and surgery prognosis), operative target, and operative stimulation projection.

### 5.2. Operative indication

Among the eight references focused on DBS, two discussed the timing of DBS, highlighting its significance as a research hotspot. The findings suggest that DBS surgery could be considered for PD patients with a disease duration of ≥5 years (or ≥3 years if severe tremor persist after standardized drug treatment), those experiencing the “on-off phenomenon” and Hoehn Yahr stage in the range of 2.5–4, or individuals aged below 75 years. However, flexibility in these restrictions is recommended based on individual patient health (FNG Chinese Society of Neurosurgery et al., 2020). Numerous studies have demonstrated that DBS surgery is beneficial for PD patients with motor complications in the middle to late stages of the disease. In contrast, Schuepbach et al. (2013) WMM’s study included PD patients with relatively early disease stages (disease duration <5 years and young age) for DBS treatment, yielding positive outcomes (Schuepbach et al., 2013). Krack et al. (2003) proposed that the procedure is more effective in patients with PD dyskinesia who respond well to medication and are relatively young. Hacker ML et al. included PD patients who had received pharmacological treatment for an average of 7.5 years and had recently developed motor complications (Hacker et al., 2015). These patients underwent DBS surgery with favorable results, suggesting that the timing of undergoing DBS surgery is advanced from patients with a long disease course to patients who present early with motor complications. After a thorough evaluation by a team of specialized physicians, patients with early stage PD and recently developed motor complications can benefit from DBS surgery.

Few studies have been conducted on older (>75 years) PD populations, and none of the highly co-cited references in this study specifically examined older patients with PD tremor. This indicates a gap in DBS research concerning aged PD patients. As a neurodegenerative disease, PD primarily affects individuals in middle and old age. However, most studies included in the current analysis and consensus recommendations for surgery focused on patients aged less than 75 years. There is a need for in-depth research on surgical interventions for PD patients older than 75 years. DeLong et al. collected and analyzed data from 1,750 patients treated with DBS between 2000 and 2009, and found that the overall complication rate did not increase with aging (DeLong et al., 2014). Some argue that performing DBS early may lead to very short disease duration, making it difficult to achieve a precise diagnosis and potentially resulting in misdiagnosis (Charles et al., 2014). On the other hand, others suggest that performing DBS very late may exacerbate primary conditions, increase post-operative complications and mortality, and impact the long-term efficacy of STN-DBS. The timing of surgery remains a subject of heated debate. Whether the constraints on disease duration and age restrictions could be relaxed, allowing DBS surgery to be performed on younger or older patients, has been a topic of significant discussion in recent years but remains uncertain.

Four out of the eight references examined in this study focused on operative targets. Schuurman et al. (2000) demonstrated the superiority of the Subthalamic Nucleus (STN) over the thalamus as a target. Deep-Brain Stimulation for Parkinson’s Disease Study Group et al. (2001) acknowledged the effectiveness and safety of two DBS targets, namely, the GPi and STN. Cury et al. (2017) highlighted the long-term effectiveness of the ventral intermediate nucleus of the thalamus (Vim) target for PD tremor without delaying PD progression. Krack et al. (2003) showed that STN-DBS significantly improved all post-operative PD motor symptoms.

### 5.3. Surgical targets

The efficacy of DBS surgery in treating PD tremor varies based on the chosen target. Among the top 10 highly co-cited references, four discussed STN, GPi, and Vim as operative targets. These three targets, STN, GPi, and Vim, have been extensively studied for PD tremor. In our investigation, STN surgery constituted over half of the clinical studies, which aligns with the prevailing trend among research teams.

Schuurman et al. (2000) and Deep-Brain Stimulation for Parkinson’s Disease Study Group et al. (2001) conducted discussions on the effects of different targets for DBS. There should be an authoritative recommendation on target for surgical treatment of tremor in Parkinson’s disease. The primary targets for PD surgical treatment are STN and GPi, which demonstrate similar improvements in motor symptoms, including tremor, in PD patients (Ramirez-Zamora and Ostrem, 2018; Wong et al., 2019). STN-DBS offers significant benefits in terms of post-operative reduction in anti-PD drug dosage (Odekerken et al., 2015). Research by Volkmann et al. (1998) reported a remarkable 65% decrease in drug use following STN stimulation, with less electrical stimulation required, leading to a more cost-effective treatment. Additionally, some animal experiments suggest that STN stimulation may also have neuroprotective effects (Wallace et al., 2007). STN-DBS is recommended for patients experiencing dopaminergic drug-induced behavioral disorders (Fasano et al., 2010; Weaver et al., 2012; Odekerken et al., 2015). However, it is crucial to consider that STN-DBS carries slightly higher surgical risks concerning cognitive function, particularly for patients with preoperative language and cognitive deficits associated with PD. In such cases, GPi-DBS is a more suitable choice. GPi provides a more cost-effective approach to alleviate and treat motor deficits, and its long-term stability and cognitive benefits offer distinct advantages (St George et al., 2014; Toft and Dietrichs, 2014).

Subthalamic Nucleus-DBS requires more post-operative modulation and has a higher incidence of adverse effects associated with levodopa withdrawal compared to GPi-DBS. On the other hand, the Vim target has proven effective and long-lasting for PD patients with significant tremor but shows limited effectiveness for other symptoms such as rigidity, bradykinesia, and drug-induced allodynia (Deuschl et al., 2006; Schuepbach et al., 2013). Additionally, a study at the University of Kansas Medical Center indicated that Vim-DBS had no significant improvement in post-operative affective cognition (Troster et al., 1998). However, Woods et al. (2001) conducted a 1-year follow-up study of six PD patients treated with Vim-DBS and found that PD patients could benefit from Vim-DBS in terms of both neurocognitive safety and quality of life. Nonetheless, further studies are necessary to confirm the role of Vim in affective cognition. Patients with relatively good motor function but disabling tremor may experience better outcomes from Vim-DBS. Table 4 provides a comparison of the advantages and disadvantages of STN, GPi, and Vim targets.

**TABLE 4:** Comparison of the advantages and disadvantages of STN, GPi, and Vim targets.

Target	Advantages	Disadvantages
STN	1. Reduced levodopa dose. 2. More comprehensive PD symptom control. 3. Lower energy consumption and higher cost performance. 4. Possible neuroprotective function	Complications of surgery include cognitive decline, psychological problems (anxiety and depression), speech, balance, and postural gait disorders, which require more medications.
GPi	1. Control tremor in PD. 2. Significantly improves motor retardation and rigidity. 3. No significant speech, psychological, or neurological damage	Inability to reduce drug dose
Vim	Highly effective for controlling tremor. Long duration of action.	1. It works only for tremor and not for other symptoms, such as rigidity and bradykinesia. 2. Bilateral stimulation can produce cognitive problems, worsening dysphonia, sensory abnormalities, gait disturbances, pain, and other adverse effects. 3. Controversy about affective cognition.

### 5.4. DBS protocols

Two references address the improvement of DBS devices. Little et al. (2013) demonstrated the design of a Brain-Computer Interface (BCI)-controlled adaptive DBS (aDBS) and optimized the stimulation protocol. Follett et al. (2010) highlighted that advancements in pulse generators can mitigate the impact of amplitude in DBS surgery. Beyond electrodes, hardware innovations in recent years have focused on pulse generators, moving toward energy-efficient and miniaturized technology. Some pulse generators can generate novel waveforms with an independent current control system (Weiss and Pal, 2018). These advancements have provided novel stimulation waveforms and modalities with temporal changes that activate corresponding neurons. By varying waveforms and pulse intervals, different stimulation modalities can be achieved. During long-term post-operative follow-up, an increasing number of PD patients experience adverse reactions or poor symptom control, leading researchers to develop innovative settings for stimulation voltage, current, polarity, pulse width, waveform, and frequency to obtain new program control strategies. Additionally, improvements in Directional Leads and pulse generators have enhanced the diversification of post-operative program control modes, aiming to achieve maximum symptom control with minimal stimulation (Aubignat et al., 2020). To explore optimal program control strategies, multiple program control technology software or algorithms can be utilized. However, no single software or algorithm can address all symptoms, necessitating further research in breakthroughs related to electrode and cell design, stimulation paradigms, closed-loop, on-demand stimulation, and sensing technologies to enhance the effectiveness and tolerability of DBS. These potential directions hold promise for future research.

### 5.5. MRgFUS

In addition, the result that the article about MRgFUS ranked the first of the top 10 most frequently co-cited references, combined with the result of the co-occurring keywords burst, indicated that, with significant research potential, MRgFUS might be a new hotspot in the field of surgical treatment of tremor in PD.

This novel non-invasive nerve destruction technique utilizes the combination of MRI and high-intensity focused ultrasound. By employing MRI for precise real-time target localization and intraoperative temperature monitoring, focused ultrasound ablation generates thermal effects on the target area *in vivo*. The technique is irreversible (Xiong and Yq, 2020).

The paper compares the treatment effects of DBS and MRgFUS for tremor in PD. DBS currently serves as the mainstream surgical approach, while MRgFUS offers advantages as a new type of nerve destruction technique. MRgFUS has demonstrated efficacy in managing significant tremor and is particularly effective in drug-refractory PD tremor (Magara et al., 2014; Schlesinger et al., 2015). It is generally considered suitable for patients with more severe unilateral symptoms of PD tremor (Martinez-Fernandez et al., 2018). Moreover, MRgFUS serves as a viable surgical option for PD patients who are not candidates for craniotomy, have contraindications to DBS, or have limited access to regular device reprogramming resources. Table 5 provides a comparison of the principles, advantages, and disadvantages of DBS and MRgFUS.

**TABLE 5:** Principles, advantages, and disadvantages of DBS and MRgFUS.

Surgery	Principle	Advantages	Disadvantages
DBS	Neuromodulatory mechanism	1. Adjustable range to increase precision. Unlimited treatment targets	1. Requires craniotomy.
		2. Multiple programmable modalities for repeated stimulation	2. Higher risk of intracranial hemorrhage and infection.
		3. Reversibility.	3. Need for long-term implantation of artificial materials.
			4. General anesthesia.
			5. Multiple post-operative procedures and device maintenance are required.
			6. DBS device implantation does not allow ultrasound or MRI.
MRgFUS	Neural destruction mechanisms	Non-invasive. No anesthesia required. No ionizing radiation. Mild adverse reactions and rare severe adverse reactions (Fishman et al., 2018).	Skin preparation required. Claustrophobia. Irregular destruction of target tissues by ultrasound ablation, with possible over- or under-destruction and risk of recurrence (Zong et al., 2020). Irreversible (Elias et al., 2013). May burn the scalp.

The paper compares the treatment effects of DBS and MRgFUS for tremor in PD. DBS currently serves as the mainstream surgical approach, while MRgFUS offers advantages as a new type of nerve destruction technique. MRgFUS has demonstrated efficacy in managing significant tremor and is particularly effective in drug-refractory PD tremor (Magara et al., 2014; Schlesinger et al., 2015). It is generally considered suitable for patients with more severe unilateral symptoms of PD tremor (Martinez-Fernandez et al., 2018). Moreover, MRgFUS serves as a viable surgical option for PD patients who are not candidates for craniotomy, have contraindications to DBS, or have limited access to regular device reprogramming resources. Table 5 provides a comparison of the principles, advantages, and disadvantages of DBS and MRgFUS.

Although MRgFUS has garnered increasing attention, it remains an emerging surgical treatment. Prospective multicenter large-scale randomized controlled trials and cohort studies are necessary to further assess its merits. In summary, MRgFUS, as a novel nerve destruction technique, can serve as a complementary surgical modality to neuromodulation and is currently a prominent area of research in the surgical treatment of tremor in PD.

### 5.6. Research trends in the surgical treatment of tremor in PD

Surgery is an indispensable treatment for tremor in PD. Our study spanning from 2002 to 2022 reveals a shift in the research hotspot within the field of surgical treatment for PD tremor. Attention and interest have transitioned from pallidotomy to DBS. Although the treatment efficacy of DBS for PD tremor is widely acknowledged, numerous and hotly debated issues and controversies persist regarding operative indications, targets, and DBS devices. Recently, MRgFUS has garnered increasing attention. Our study also provides a comprehensive comparison of the advantages and disadvantages between DBS and MRgFUS.

This study presents several notable advantages. Firstly, this study represents a pioneering effort as the first bibliometric and visualization analysis conducted in the field of surgical treatment of tremor in PD, demonstrating significant innovation. Secondly, our study utilizes the WOS database, a prominent tool in bibliometric analysis, for objective and systematic retrieval. Thirdly, we primarily employ a novel bibliometric software, Citespace, which provides a fresh approach to elucidating the underlying mechanisms of various changes and patterns in scientific networks. By identifying critical nodes based on objective data, we can discern the research hotspots in surgical treatment for PD tremor, thus offering valuable directions for future clinical practices and research in this field. Finally, our data analysis is comprehensive and rigorous, encompassing various aspects such as annual publication volume, authors, institutions, countries/regions, and co-cited literature. This in-depth analysis of surgical treatment for PD tremor enables readers to gain a comprehensive understanding of peer education, training, and scientific research guidance.

This study also exhibits some limitations. Firstly, it solely searched the English literature in the WoS database, limiting the scope to the years 2002 to 2022. Consequently, the selected keywords might not fully represent all relevant studies in the field, potentially introducing bias. Additionally, considering that surgery for PD tremor encompasses a vast area, a more comprehensive understanding of specific surgical aspects may be necessary for this study.

## 6. Conclusion

This research employs a comprehensive and systematic bibliometric and visualization analysis to visualize the evolution of research and emerging hotspots. The identified hotspots are as follows: Firstly, DBS has garnered considerable attention and widespread recognition in the surgical treatment of PD tremor. Secondly, there are numerous critical issues in operative indications, operative targets, and surgical protocols related to DBS. Thirdly, MRgFUS has emerged as a promising treatment in the field of surgical treatment for PD tremor. This study also elucidates these hotspots, providing valuable insights and guidance for future research.

## Data availability statement

The raw data supporting the conclusions of this article will be made available by the authors, without undue reservation.

## Author contributions

JCZ and HC: conceptualization, writing-original draft, and formal analysis. YQL and XX: methodology, software, and validation. LML: visualization and investigation. JJL: conceptualization and supervision. GAL: data curation. LSL and LHL: writing—review and editing. NGX and SXW: project administration and funding acquisition. All authors contributed to the article and approved the submitted version.
