# Supplementary material for: Research status and hotspots in the surgical treatment of tremor in Parkinson’s disease from 2002 to 2022: a bibliometric and visualization analysis
Source: Front Aging Neurosci. 2023 Sep 27;15:1157443. doi: 10.3389/fnagi.2023.1157443 (PMC10565824; doi:10.3389/fnagi.2023.1157443)
Supplement: Supplementary file 1 [file Data_Sheet_1.docx]

Supplementary Material

Research Status and Hotspots in the Surgical Treatment of Tremors in Parkinson’s Disease from 2002–2022: A Bibliometric and Visualization Analysis

Jingchun Zeng1†, Hui Chu2†, Yiqian Lu2, Xi Xiao1, Liming Lu3, Jingjing Li4, Guoan Lai2, Lisha Li5, Lihong Lu1, Nenggui Xu3, Shuxin Wang1*

†These authors contributed equally to this work and share first authorship

*** Correspondence:** Shuxin Wang*,widiot@126.com

# Supplementary Figures and Tables

## Supplementary Figures

##
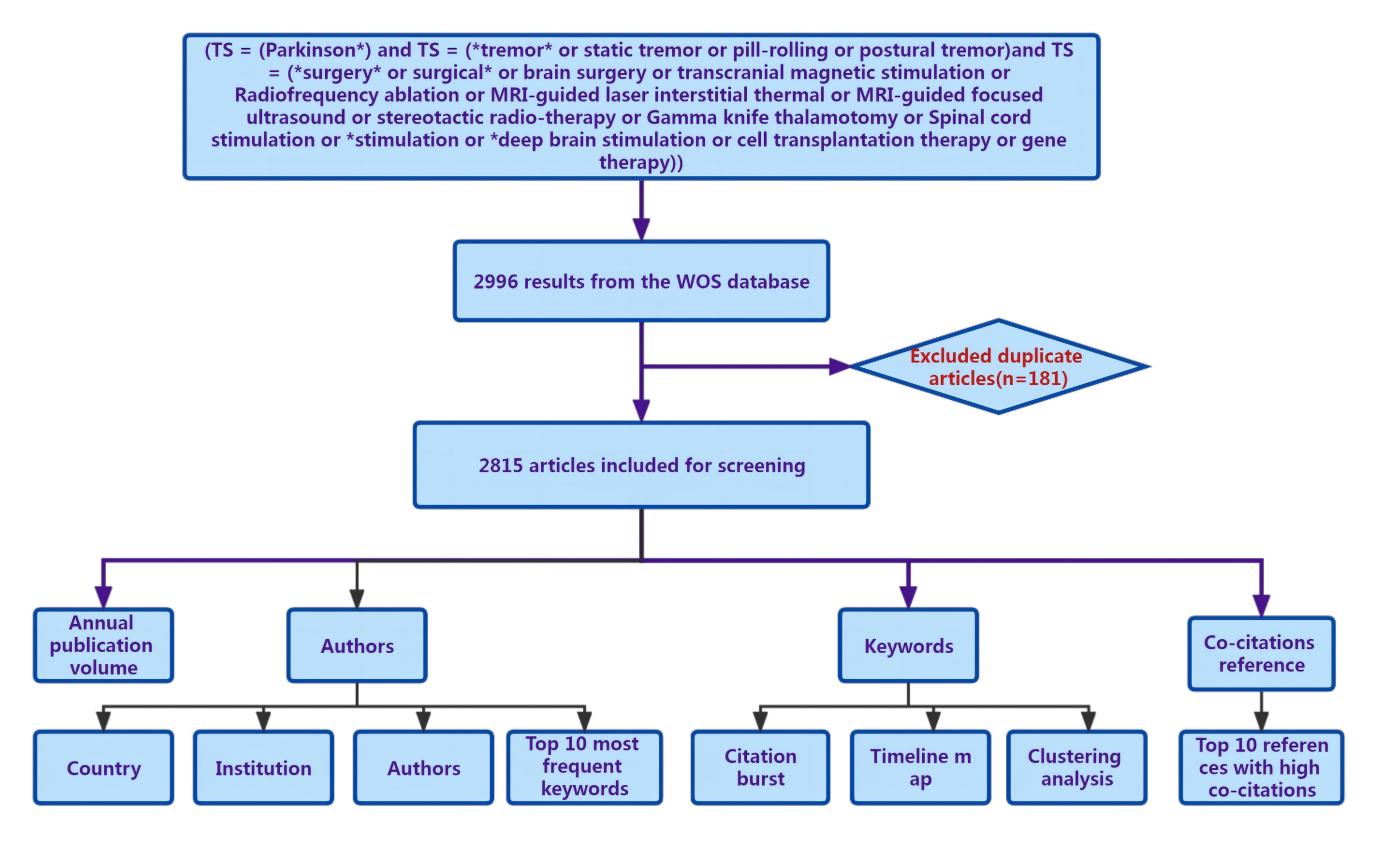


## Supplementary Figure 1. Flowchart of the selection process for the eligible literature.


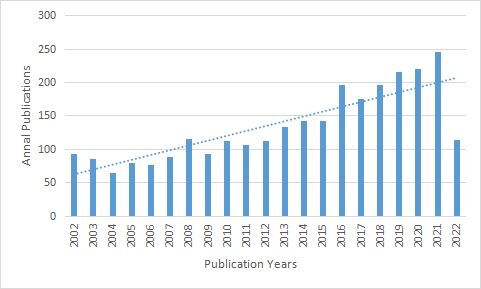


## Supplementary Figure 2. Number of annual publications and growth trends.


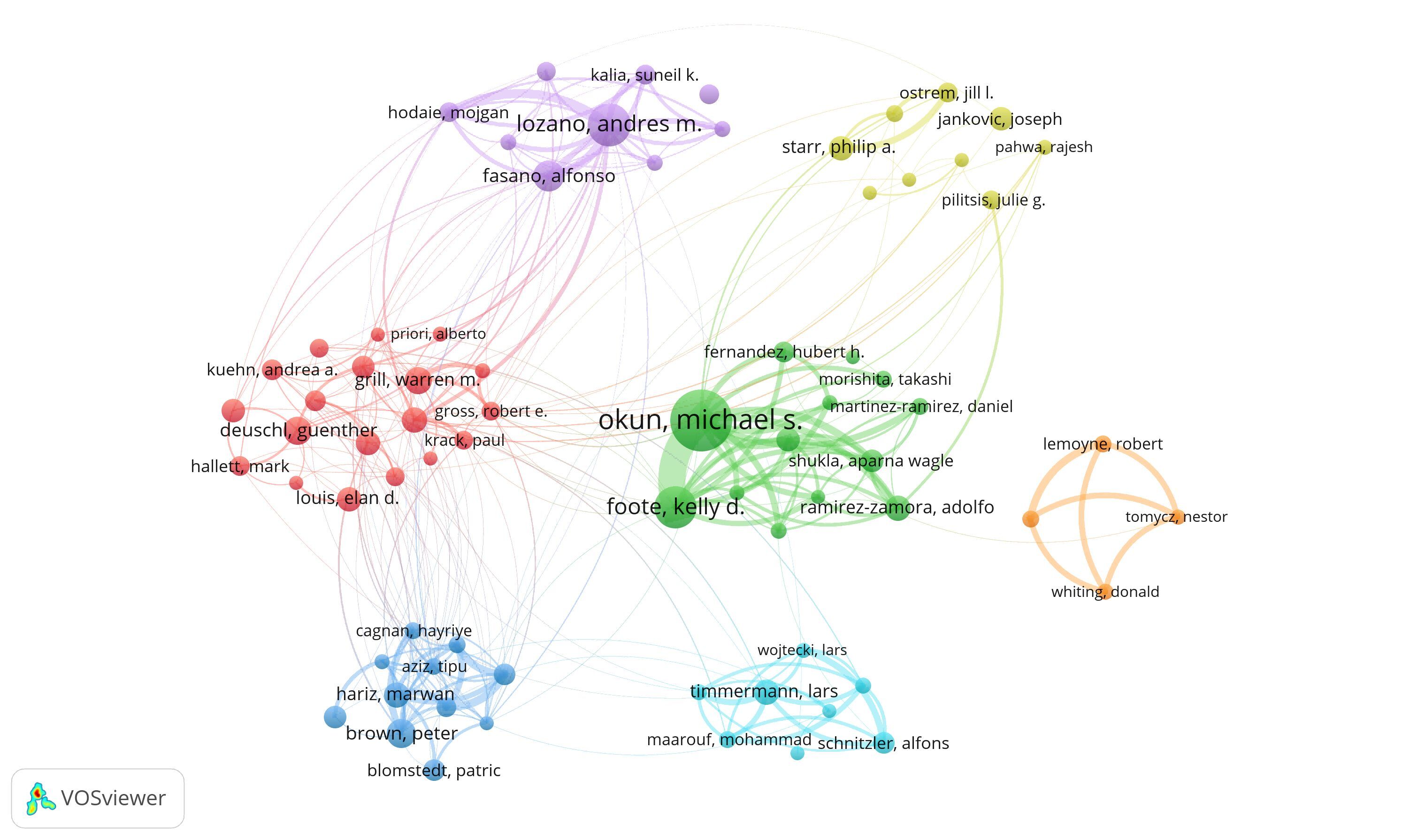


## Supplementary Figure 3. Network map of authors from VOSviewer.


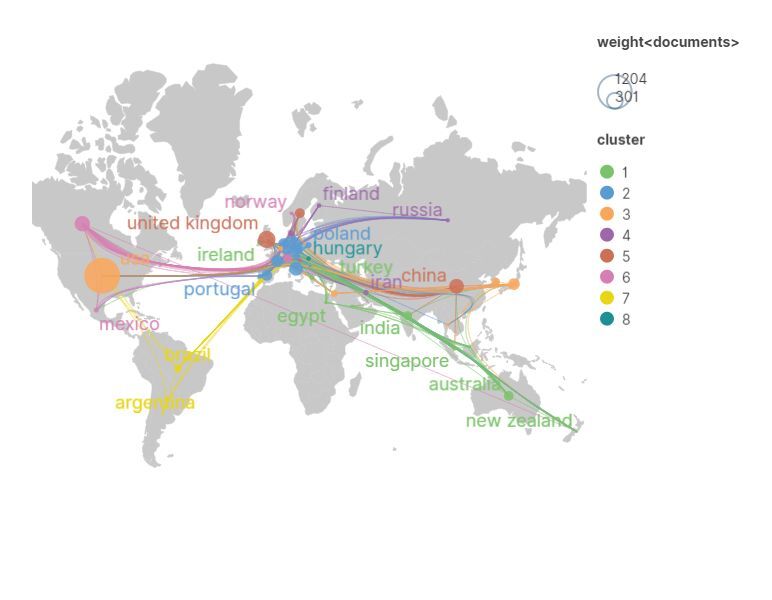


## Supplementary Figure 4. Global geographical distribution of surgical treatment of tremors in PD.


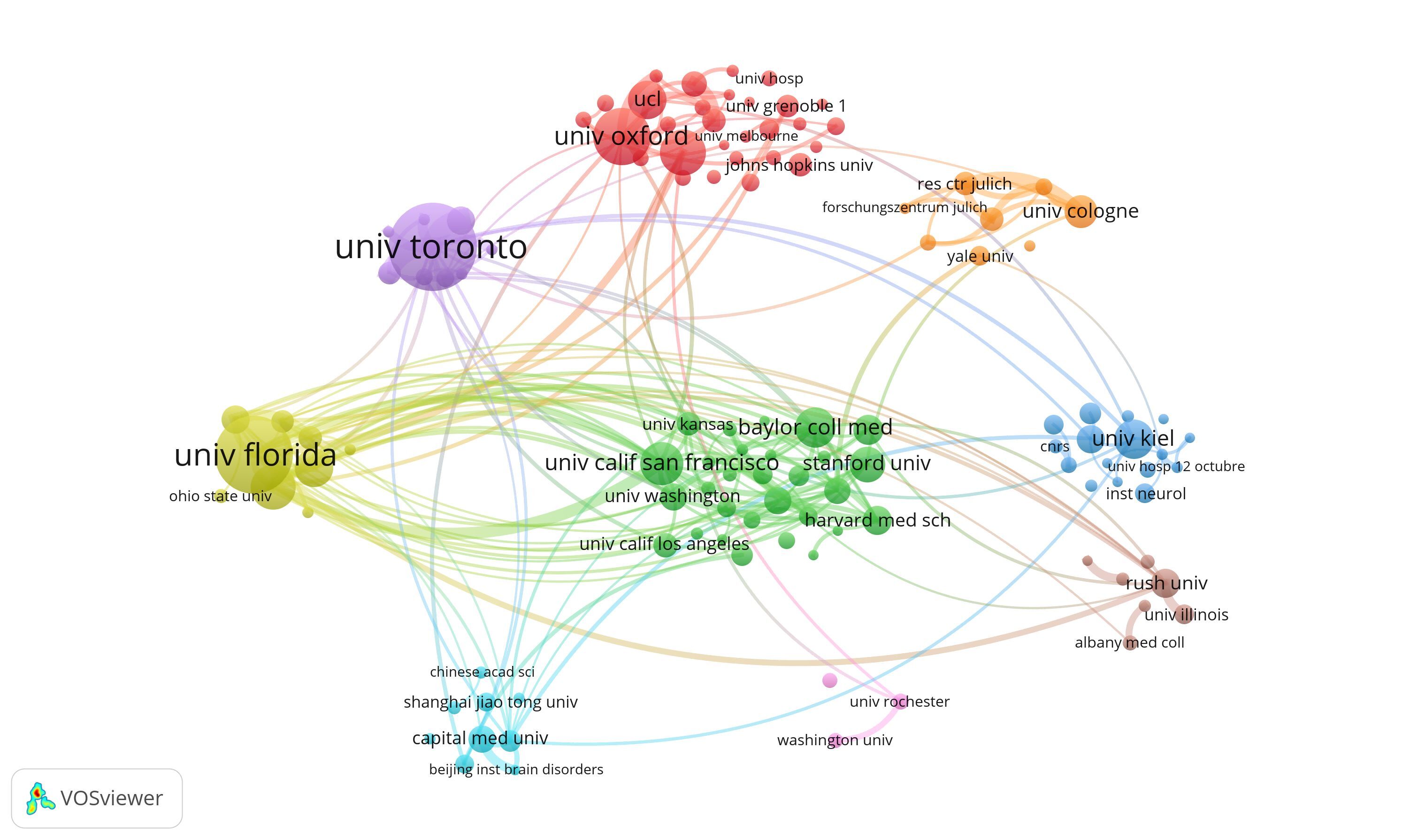


## Supplementary Figure 5. Network map of institutions from VOSviewer.


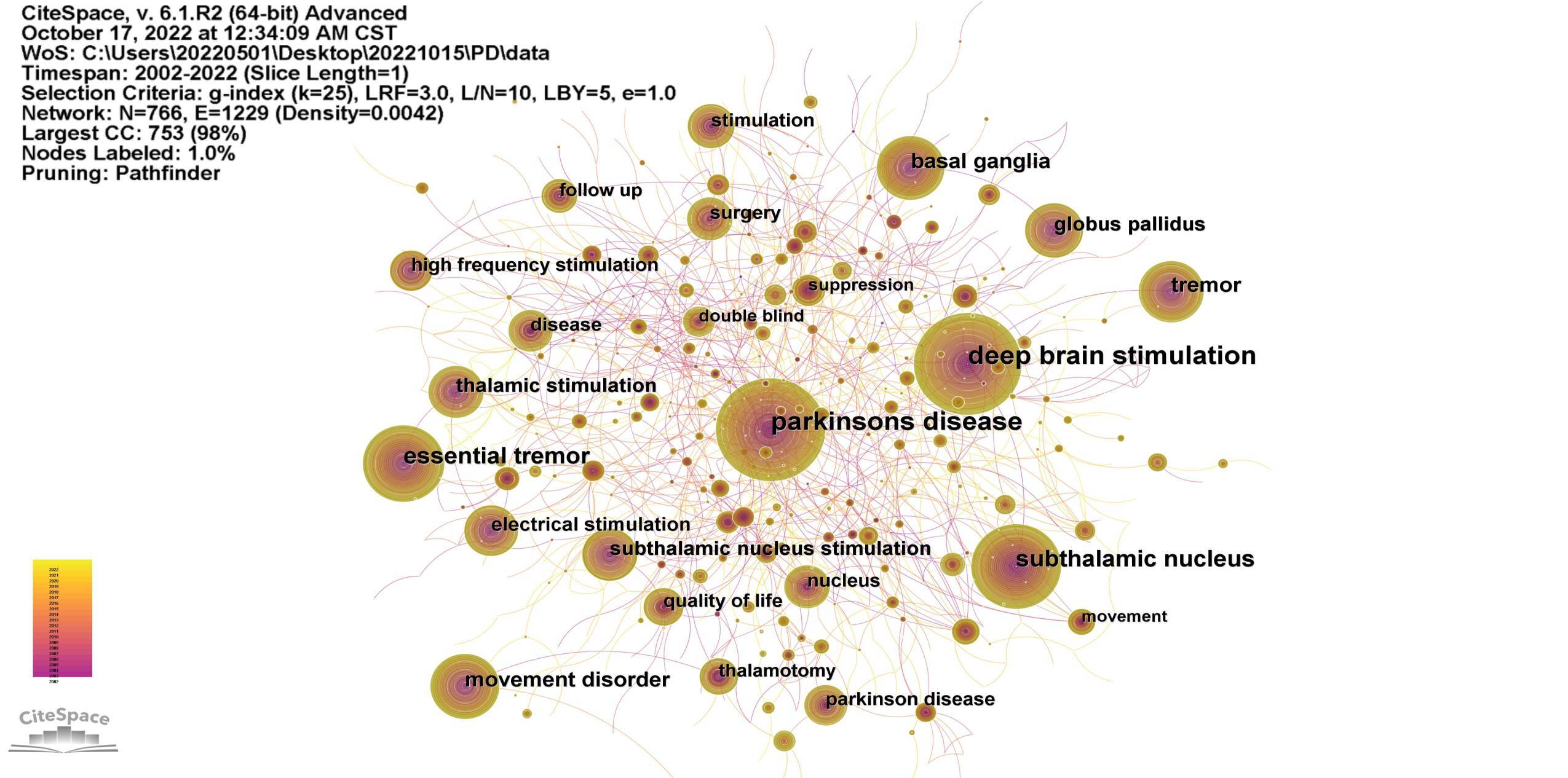


## Supplementary Figure 6. Network map of co-occurring keywords from CiteSpace.


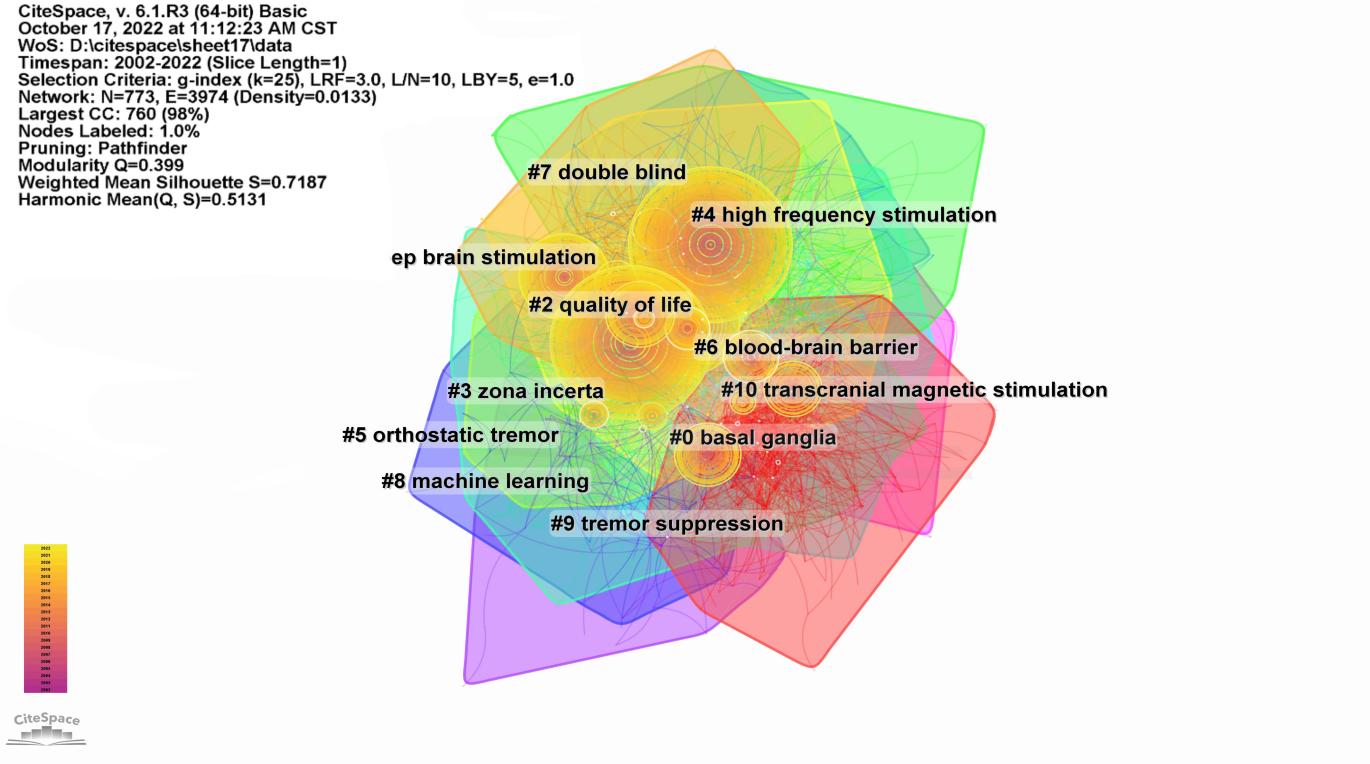


## Supplementary Figure 7. Clustering map of co-occurring keywords from CiteSpace.


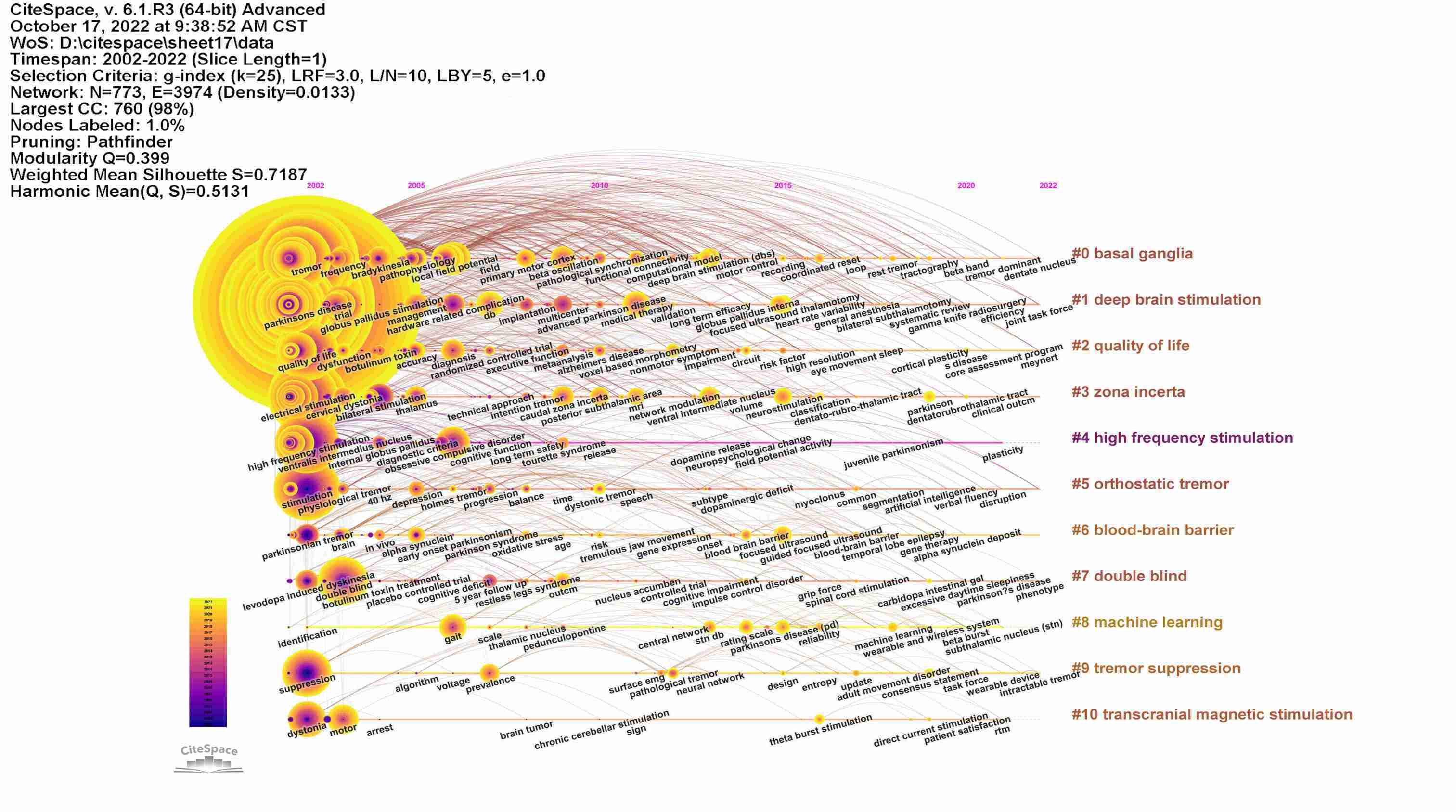


## Supplementary Figure 8. Timeline map of co-occurring keywords from CiteSpace.


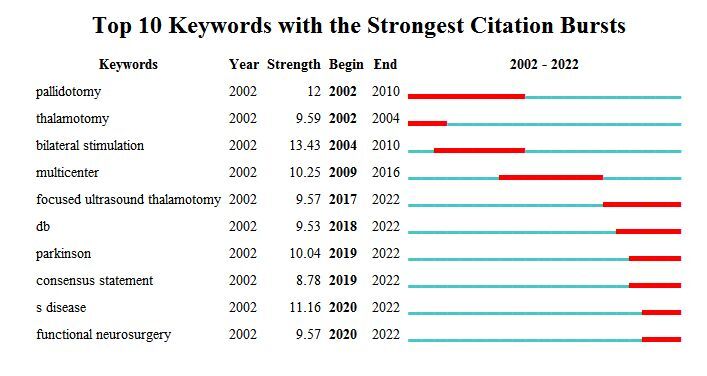


## Supplementary Figure 9. Top 10 keywords with the strongest citation bursts.


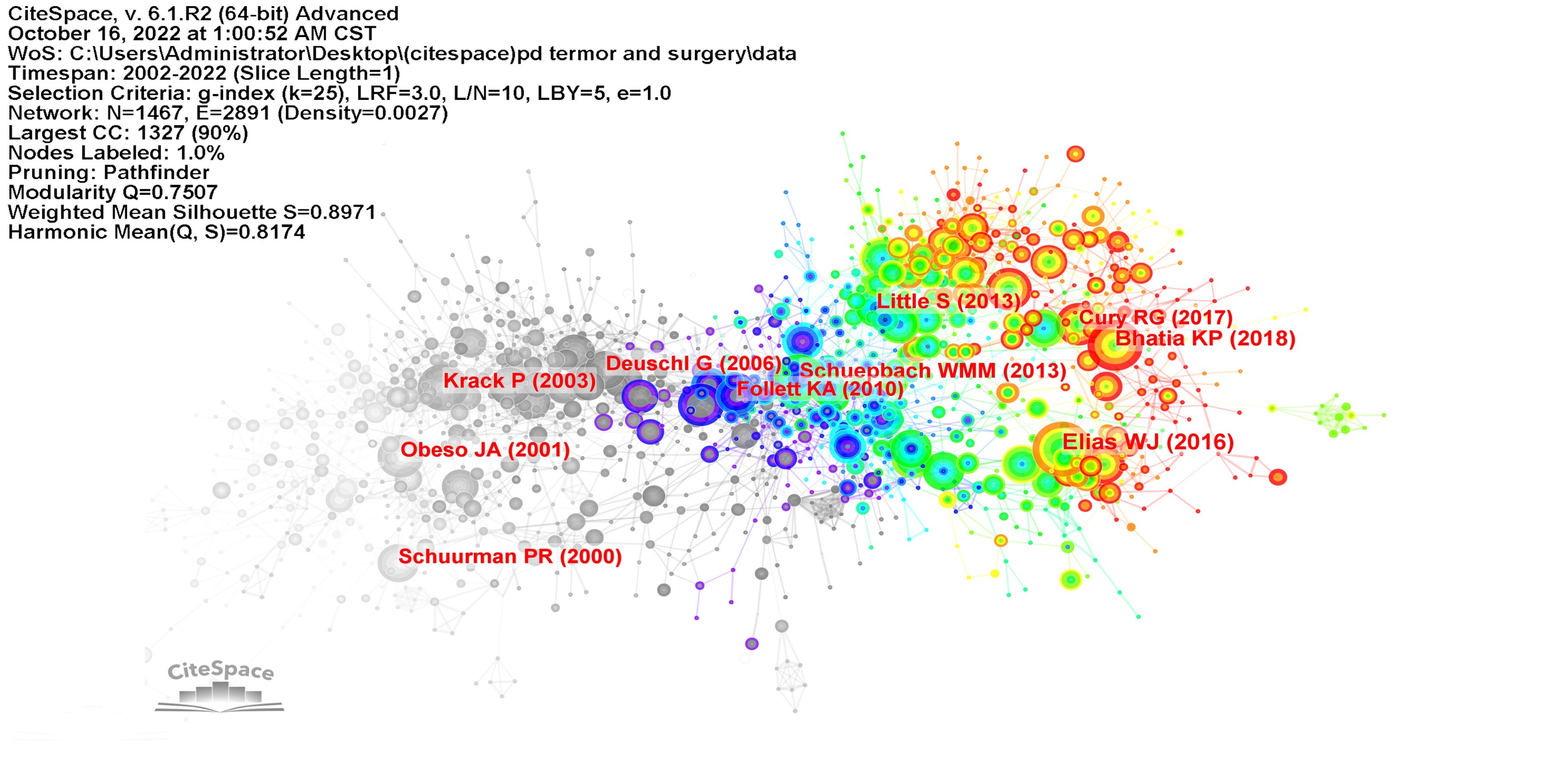


## Supplementary Figure 10. Network map of co-citations from CiteSpace.

## Supplementary Tables

1. **Tables**

# Tables

**Table 1.** Top 10 countries/regions by publications

| **Rank** | **Countries/regions** | **Publications** | **Percentage (total of 2,815; %)** |
| --- | --- | --- | --- |
| 1 | USA | 1204 | 42.8 |
| 2 | Germany | 400 | 14.2 |
| 3 | United Kingdom | 279 | 10.0 |
| 4 | Canada | 221 | 7.9 |
| 5 | China | 204 | 7.2 |
| 6 | Italy | 178 | 6.3 |
| 7 | France | 139 | 4.9 |
| 8 | Japan | 129 | 4.6 |
| 9 | Netherlands | 116 | 4.1 |
| 10 | Spain | 109 | 3.9 |

**Table 2.** Top 10 institutions according to publications.

| **Rank** | **Publications** | **Institutions** | **Original country** |
| --- | --- | --- | --- |
| 1 | 128 | University of Toronto | Canada |
| 2 | 111 | University of Florida | United States |
| 3 | 78 | University of Oxford | England |
| 4 | 59 | Mayo Clinic | United States |
| 5 | 57 | Emory University | United States |
| 6 | 54 | University of California San Francisco | United States |
| 7 | 50 | Baylor College of Medicine | United States |
| 8 | 49 | University of Kiel | Germany |
| 9 | 48 | University College London | United States |
| 10 | 48 | Duke University | United States |

**Table 3.** Top 10 highly cited references details.

| **First author (year)** | **Citation counts** | **Journal** | **Design or type of study** | **Sample size** | **Participants** | **Intervention** | **Outcomes** | **Highlights** |
| --- | --- | --- | --- | --- | --- | --- | --- | --- |
| Elias WJ (2016) | 109 | *NEJM* | Single-blind, randomized, controlled trial | 76 | Patients diagnosed with essential tremor, with moderate to severe postural or intentional tremor of the hand, who have failed at least two trials of drug therapy and whose current drug dose is stable for primary tremor. | MRgFUS vs. sham procedure. | The Clinical Rating Scale for tremor and the Quality of Life in Essential tremor Questionnaire | 1. Demonstrated the efficacy of FUS in drug-refractory primary tremor. 2. Long-lasting efficacy (12 months) |
| Bhatia KP (2018) | 97 | *Movement Disorders* | Consensus | \ | \ | \ | \ | 1. Classification of tremor based on two axes.  2. To provide a framework for clinical diagnosis of new tremor syndromes for further etiological studies. |
| Krack P (2003) | 79 | *NEJM* | Follow-up study | 49 | Patients aged less than 70 years with advanced PD after bilateral stimulation of the subthalamic nucleus | Bilateral STN-DBS | UPDRS, Mattis Dementia Rating Scale, an assessment of frontal-lobe dysfunction, Beck depression  inventory | 1. Demonstrated that STN-DBS significantly improved all postoperative PD motor symptoms. 2. No stimulus tolerance was found (5-year follow-up). 3. It is proposed that the procedure is more effective in patients with PD dyskinesia who respond well to medication and are relatively young. |
| Little S (2013) | 78 | *Annals of Neurology* | Randomized controlled study | 8 | Patients with advanced idiopathic PD  with motor fluctuations and/or dyskinesias | aDBS vs. no stimulation vs. cDBS. | UPDRS | 1. Optimize the DBS stimulation protocol. 2. Design a BCI-controlled adaptive DBS (aDBS). |
| Schuepbach WMM (2013) | 77 | *NEJM* | Randomized,  multicenter, parallel-group | 251 | PD patients with early motor complications | DBS plus medical therapy or medical therapy alone | UPDRS, the time with good mobility and no dyskinesia. | Neurostimulation was indicated for earlier, younger patients (mean duration of disease of 7.5 years; mean age of 52 years; fluctuations and motor deficits for 1.7 and 1.5 years, respectively). |
| Deuschl G (2006) | 71 | *NEJM* | Non-blind, randomized, paired trial | 156 | Patients younger than 75 years with impaired motor symptoms of PD affecting the ability to perform daily living with optimal drug therapy. | DBS group vs. drug group | PDQ-39, UPDRS | 1. Used quality of life as the primary outcome indicator  2. The risks of surgery need to be weighed against the benefits of surgery. |
| Schuurman PR (2000) | 70 | *NEJM* | Randomized controlled trial | 68 | Severe unilateral or bilateral arm tremor due to PD, primary tremor, or multiple sclerosis for at least one year in the case of medication. | Thalamotomy or thalamic stimulation | Frenchay Activities Index,  UPDRS, Essential tremor Rating Scale, Modified Tremor Scale. | 1. DBS is more effective and safer than thalamotomy.  2. STN is a superior target than thalamus. |
| Obeso JA (2001) | 69 | *NEJM* | Prospective, double-blind, crossover study | 134 | Patients aged 30–75 years with at least two major features of PD (tremor, rigidity, and bradykinesia) that are not controlled by medication and respond well to levodopa | Randomly turn on/off the DBS to STN or globus pallidus(GPi) | UPDRS, a dyskinesia-rating scale, a home diary | 1. Demonstrated the effectiveness and safety of two DBS targets .  2. STN may be superior to the pallidum as a target. |
| Cury RG (2017) | 65 | *Neurology* | Follow-up study | 98 | PD, ET, and dystonia due to refractory tremor | VIM-DBS | UPDRS; Fahn, Tolosa, Marin tremor Rating Scale | 1. Long follow-up period (over 10 years)  2. VIM is effective for PD tremor in the long term but does not delay PD progression.  3. VIM is the preferred target for tremor. |
| Follett KA (2010) | 65 | *NEJM* | Multi-center, randomized, blinded trial | 299 | PD patients over 21 years of age with Hoehn Yahr score greater than 2 who are stable on medication and have poor efficacy | Pallidal DBS or STN- DBS | Hoehn and Yahr scale, Schwab and  England scale of activities of daily living, stand–walk–sit test; UPDRS, PDQ-39, Beck depression inventory-II. | 1. Demonstrated the efficacy of DBS in the two target areas and the reduction of dopaminergic medication.  2. Improvements in pulse generators can reduce the impact of amplitude in DBS surgery. |

**Table 4.** Comparison of the advantages and disadvantages of STN, GPi, and Vim targets

| **Target** | **Advantages** | **Disadvantages** |
| --- | --- | --- |
| STN | 1.Reduced levodopa dose.  2.More comprehensive PD symptom control.  3.Lower energy consumption and higher cost performance.  4.Possible neuroprotective function | Complications of surgery include cognitive decline, psychological problems (anxiety and depression), speech, balance, and postural gait disorders, which require more medications. |
| GPi | 1. Control tremor in PD.  2. Significantly improves motor retardation and rigidity.  3. No significant speech, psychological, or neurological damage | Inability to reduce drug dose |
| Vim | 1. Highly effective for controlling tremor. 2. Long duration of action. | 1. It works only for tremor and not for other symptoms, such as rigidity and bradykinesia.  2. Bilateral stimulation can produce cognitive problems, worsening dysphonia, sensory abnormalities, gait disturbances, pain, and other adverse effects.  3. Controversy about affective cognition. |

**Table 5.** Principles, advantages, and disadvantages of DBS and MRgFUS

| **Surgery** | **Principle** | **Advantages** | **Disadvantages** |
| --- | --- | --- | --- |
| DBS | Neuromodulatory mechanism | 1.Adjustable range to increase precision. Unlimited treatment targets  2. Multiple programmable modalities for repeated stimulation  3. Reversibility. | 1.Requires craniotomy.  2.Higher risk of intracranial hemorrhage and infection.  3.Need for long-term implantation of artificial materials.  4.General anesthesia.  5.Multiple post-operative procedures and device maintenance are required.  6.DBS device implantation does not allow ultrasound or MRI. |
| MRgFUS | Neural destruction mechanisms | Non-invasive. No anesthesia required. No ionizing radiation. Mild adverse reactions and rare severe adverse reactions[51]. | Skin preparation required. Claustrophobia. Irregular destruction of target tissues by ultrasound ablation, with possible over- or under-destruction and risk of recurrence[52]. Irreversible[53]. May burn the scalp. |
